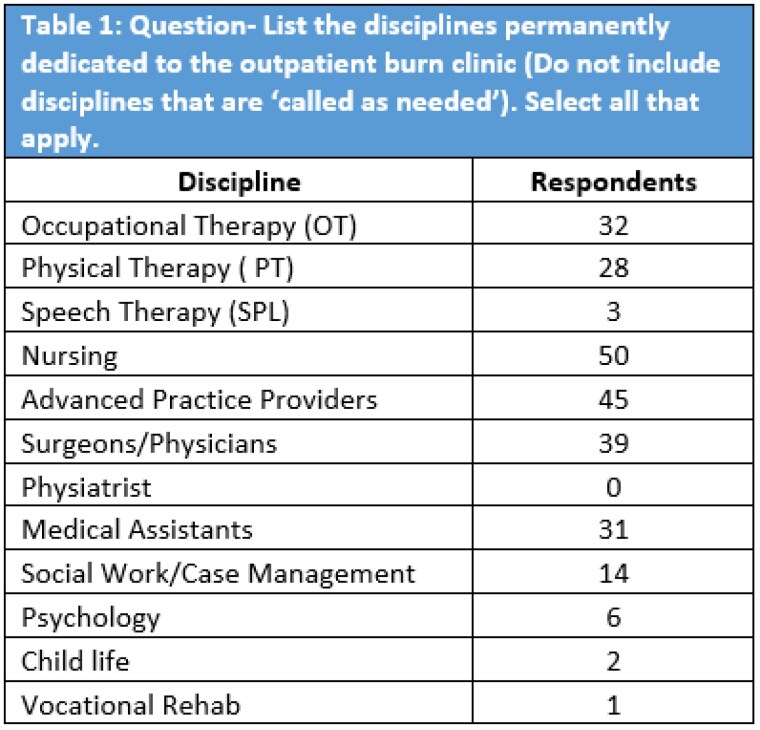# 68 Current Status of Therapy Utilization Within the Outpatient Burn Setting

**DOI:** 10.1093/jbcr/iraf019.068

**Published:** 2025-04-01

**Authors:** Audrey O’Neil, Ingrid Parry, Derek Murray, Soman Sen, Brett Hartman

**Affiliations:** Richard M. Fairbanks Burn Center; Shriners Children’s Northern California; Diane & Bruce Halle Arizona Burn Center; University of California, Davis and Shriners Children’s; Eskenazi Health

## Abstract

**Introduction:**

Point of care for burn survivors has traditionally been the inpatient burn unit. Advances in burn care have resulted in significantly reduced length of stay or allowed survivors to avoid admission. However, an outpatient shift within burn care only emphasizes a growing need for rehabilitation resources. This study surveyed burn centers within the United States (US) to determine current outpatient burn clinic structure and evaluate utilization of burn therapy.

**Methods:**

A 20-question survey was electronically distributed to burn clinicians at burn centers within the US. Questions included the location of their burn clinic, operational hours, dedicated staff, and utilization of burn therapy services. Chi-square test was used to analyze frequency differences between American Burn Association (ABA) verified and non-verified centers with significance set at < 0.05.

**Results:**

The survey was completed by representatives of 54 burn centers treating Adults (n=17), Pediatric (n=7), or both (n=30). Burn centers were ABA verified (61%, n=33) and non-verified (39%, n=21) centers. Outpatient clinics included permanently dedicated burn therapists at 65% (n=35) of responding burn centers (OT= 32, PT =28) (Table 1). Respondents reported that therapy services in the outpatient clinics saw “Almost all patients,” 15% (n=8) of the time, while the majority stated therapist saw “Most” (30%, n=16) or “Some” patients (24%, n=13). Only 7 centers report standing therapy orders, while the majority report pre-screening by APPs/physicians (n=43). Only 39% (n=21) reported that reconstructive patients were “Often” referred for therapy. The greatest barriers to outpatient follow up were “distance from burn center”(n=43) and “transportation”(n=30), with 50% of respondents offering transportation support for patients. Verified burn centers were statistically more likely to have either PT or OT dedicated to the burn clinic (79%, p=0.007) than non-verified centers. However, there was no difference in verification status for therapy treating “Most” or “All” patients (50%, p=0.112), and having a dedicated burn therapy treatment space (59%, p=0.067)

**Conclusions:**

Outpatient Burn Rehabilitation is currently being underutilized, with many centers setting standards below those established for inpatient burn care. Transitioning care to the outpatient setting does not eliminate, or reduce patients’ need for specialized burn therapy. Hospital, physician, and outpatient leaders must consider the rehabilitative needs of burn patients as care is shifted to outpatient and ensure adequate therapy involvement in care.

**Applicability of Research to Practice:**

Burn centers must prepare to treat an increasing number of burn survivors throughout the entire burn continuum of care. Therapy resources must be optimized within the outpatient setting to match the shift in care toward the ambulatory environment to adequately support burn survivors.

**Funding for the Study:**

N/A